# The *Drosophila* estrogen-related receptor promotes triglyceride storage within the larval fat body

**DOI:** 10.1016/j.jlr.2025.100815

**Published:** 2025-04-25

**Authors:** Tess D. Fasteen, Melody R. Hernandez, Robert A. Policastro, Maria C. Sterrett, Gabriel E. Zenter, Jason M. Tennessen

**Affiliations:** Department of Biology, Indiana University, Bloomington, IN

**Keywords:** *Drosophila melanogaster*, lipidomics, estrogen-related receptor, metabolism

## Abstract

The estrogen-related receptor (ERR) family of nuclear receptors serves key roles in coordinating triglyceride (TAG) accumulation with juvenile growth and development. In both insects and mammals, ERR activity promotes TAG storage during the postembryonic growth phase, with loss-of-function mutations in mouse *Esrra* and *Drosophila melanogaster dERR* inducing a lean phenotype. However, the role of insect ERRs in controlling TAG accumulation within adipose tissue remains poorly understood, as nearly all transcriptomic and metabolomic studies have relied on whole animal analyses. Here, we address this shortcoming by using tissue-specific approaches to examine the role of dERR in regulating lipid metabolism within the *Drosophila* larval fat body. We find that dERR autonomously promotes TAG accumulation within fat body cells and regulates expression of genes involved in glycolysis, β-oxidation, and isoprenoid metabolism. As an extension of these results, we not only discovered that *dERR* mutant fat bodies exhibit decreased expression of known dHNF4 target genes but also found that dHNF4 activity is decreased in *dERR* mutants. Overall, our findings indicate that dERR plays a multifaceted role in the larval fat body to coordinate lipid storage with carbohydrate metabolism and developmental growth.

Growth and development must be closely coordinated with metabolism to fulfill the differential energetic requirements of each life stage (1, 2). Among the key metabolites that dictate both growth rate and developmental progression are lipids, which serve in developmental signaling pathways, act as building blocks for membranes and other cellular structures, and function as energetic reservoirs that support major developmental events and ensure survival during bouts of stress and starvation (3, 4, 5, 6, 7). As a result, disruptions in lipid metabolism can significantly alter growth trajectories, adult lifespan, and reproductive capacity. For example, in humans, body fat percentage is more closely linked with the onset of mammalian puberty than chronological age (8, 9, 10). Similarly, maternal obesity disrupts metabolic cues during fetal development, resulting in altered hormone levels, decreased adipogenesis, induction of lipolysis, and precocious onset of puberty in the offspring (11, 12). Abnormal lipid metabolism, however, not only influences developmental progression but also results in increased risk of developing metabolic disorders later in life, including type 2 diabetes, heart disease, strokes, osteoarthritis, and other comorbidities (13, 14, 15). Thus, the molecular mechanisms that coordinate lipid metabolism with growth and development broadly influence animal health and disease progression.

The fruit fly *Drosophila melanogaster* has emerged as a powerful genetic system for studying the coordinate regulation of lipid metabolism and juvenile growth (16, 17, 18, 19). During the course of larval (juvenile) development, the fly accumulates large triglyceride (TAG) reserves that serve diverse and essential biosynthetic and energetic functions (16, 20, 21). Not only do these TAG stores buffer larval development against bouts of nutrient deprivation (22, 23), but larval fat tissue also persists throughout metamorphosis into the early adult stages, where TAG supports the energetic and biosynthetic requirements of newly eclosed adults (20, 24, 25). Moreover, TAG serves as a carbon sink during periods of nutrient excess, preventing sugar and lipid intermediates from accumulating to levels that induce metabolic disease phenotypes (26, 27).

The insect fat body is one of the key tissues responsible for coordinating insect TAG metabolism with larval growth and development. In its role as the functional equivalent of both mammalian liver and adipose tissue, the fat body monitors the systemic levels of lipids and other metabolites, controls the balance between macromolecular storage and interorgan metabolite transport, and produces secreted factors that regulate systemic growth (28, 29, 30, 31, 32). Considering that many of the nutrient sensors and signal transduction cascades that regulate metabolism within the larval fat body are highly conserved, this organ represents a powerful model for studying how metabolism, and lipid metabolism in particular, is regulated in the context of animal development.

Among the conserved factors that function within the fat body, dERR, the sole *Drosophila* ortholog of the estrogen-related receptor (ERR) family of nuclear receptors (NRs), stands out as a promising candidate for examining how lipid metabolism and developmental progression are coordinately regulated. Although insect ERRs primarily regulate genes involved in carbohydrate metabolism (33, 34, 35, 36), studies in both *D. melanogaster* and the mosquito *Aedes aegypti* demonstrate that loss of ERR activity results in a lean phenotype (34, 35, 36). Similarly, while mutant mice lacking ERRα exhibit relatively normal development, these animals are lean, exhibit defects in lipid uptake, and are resistant to diet-induced obesity (37, 38). Such findings not only indicate that the ERR family members play a central role in coordinating lipid metabolism with juvenile development but also position *D. melanogaster* as an ideal model for understanding how this NR family member functions within adipose tissue.

Despite the clear potential of studying dERR in adipose cells, previous studies encountered several obstacles that limit our understanding of this NR in fly TAG metabolism. Notably, dERR function has largely been analyzed using whole animal multiomic assays that potentially overlooked fat body–specific dERR functions (34, 35, 36). For example, studies of *Drosophila* larvae have previously shown that *dERR* mutants exhibit a global decrease in the expression of genes involved in glycolysis, the pentose phosphate pathway, and other aspects of carbohydrate metabolism (35). As a result, *dERR* mutants are unable to properly utilize dietary carbohydrates and die near the end of the second larval instar with elevated levels of circulating sugars and decreased TAG stores (35). This earlier study, however, failed to examine how loss of dERR activity influences gene expression or TAG accumulation specifically within the fat body. Similarly, while adult studies demonstrated that depletion of dERR transcripts using RNA interference (RNAi) in adult fat cells results in decreased TAG accumulation, the corresponding metabolomic and transcriptomic analyses of adult dERR function relied on whole animal extracts (36). Considering that a recent study of *Aedes aegypti* ERR fat body function found that this NR plays a broader role in regulating carbohydrate and lipid metabolism than previously described in other insects (34), additional studies of dERR in fat body cells are warranted. Here, we address this knowledge gap in the fruit fly by examining how dERR influences lipid metabolism specifically within the *Drosophila* larval fat body.

Using a combination of quantitative lipidomics and tissue-specific genetic manipulations, we demonstrate that dERR acts within the larval fat body to promote TAG storage. Not only does restoration of dERR expression in the fat body of *dERR* mutants rescue the previously described lean phenotype, but fat body-specific dERR-RNAi also reduced TAG accumulation within fat body cells. As a complement to these biochemical and genetic studies, we also used RNA-seq to quantify gene expression within fat bodies isolated from *dERR* mutants. This analysis revealed that *dERR* mutant fat bodies not only display a significant decrease in glycolytic gene expression but also show significant changes in the expression of genes involved in both lipid synthesis and lipid catabolism. Intriguingly, this RNA-seq dataset demonstrated that loss of dERR activity results in a significant downregulation of genes associated with fatty acid β-oxidation, a result that has not been previously observed in whole animal RNA-seq studies. Finally, we demonstrate that the NR dHNF4 displays decreased activity in *dERR* mutants. Considering that dHNF4 is a key regulator of fatty acid β-oxidation in fly larvae (39), our findings inform a model in which these highly conserved NRs function in the fat body to coordinately regulate aspects of lipid metabolism within the context of larval growth.

## Materials and Methods

### Drosophila husbandry and genetics

*Drosophila* stocks were maintained on Bloomington Stock Center medium at 25°C. *dERR* mutant larvae were generated by crossing *w*^*1118*^*; ERR*^*1*^*/TM3, P{w[+mC] = GAL4-twi.G}2.3, P{UAS-2xEGFP}AH2.3, Sb*^*1*^ *Ser*^*1*^ (RRID:BDSC_83688) virgin females with either *w*^*1118*^ males, *w*^*1118*^*; ERR*^*2*^*/TM3, P{Dfd-GMR-nvYFP}3, Sb*^*1*^ males (RRID:BDSC_83689), or *w*^*1118*^*; Df(3L)Exel6112, P{w[+mC] = XP-U}Exel6112/TM6B, Tb*^*1*^ (RRID:BDSC_7591). Offspring were collected on molasses plates covered with yeast paste as previously described (40). The resulting larvae were raised on the collection plates for 60 h at 25°C. L2 larvae of the genotypes *w*^*1118*^*; ERR*^*1/2*^ and *w*^*1118*^; and *ERR*^*1/+*^ were collected based on the absence of GFP and YFP expression. *UAS-transgene* expression was driven using *y*^*1*^ *w∗; P{w[+mC] = r4-GAL4}3* (RRID:BDSC_33832). Fat body–specific dERR rescue experiments were conducted using a previously described strain harboring a *UAS-dERR* transgene (35). RNAi experiments were conducted using *y*^*1*^ *v*^*1*^*; P{y + t7.7v[+t1.8] = TRiP.HMC03087}attP2 (RRID:BDSC_50686)*, which was generated by the *Drosophila* Transgenic RNAi Project (41). The strain *w*^*1118*^*; PBac{y[+mDint2] w[+mC] = ERR-GFP.FSTF}VK00037* (RRID:BDSC_38638) was used to examine dERR expression in the larval fat body. This strain harbors an integrated bacterial artificial chromosome that contains the *dERR* genomic locus with a GFP-StrepII-FLAG TAG inserted at the dERR C terminus (42). dHNF4-ligand-binding domain (LBD) activation experiments were conducted using the previously described *hs-GAL4-dHNF4* and *UAS-nlacZ* transgene (43). Flybase was used as a reference throughout the study (44).

### Immunofluorescence

Visualization of the dERR-GFP-StrepII-Flag protein was conducted by first dissecting larvae in 1x PBSand fixing for 30 min in 4% paraformaldehyde in 1x PBS at room temperature. Fixed tissues were then washed three times for 5 min each with PBS with 0.1% Triton X-100 (PT) and blocked for 30 min in PT with 5% normal goat serum (NGS-PT) at room temperature. Blocked tissues were incubated overnight at 4°C with Mouse anti-FLAG (# MA1-91878 Thermo Fisher Scientific) diluted 1:1000 in NGS-PT. Tissues were subsequently washed three times for 5 min each with PT and then incubated overnight with Alexa Fluor 488 Goat anti-Mouse secondary antibody (#A11029 Thermo Fisher Scientific) diluted 1:1000 in PT at 4°C. Tissues were then washed three times for 5 min each in PT and mounted in Vectashield with 4',6-diamidino-2-phenylindole (Vector Laboratories; H-1200-314 10). Stained tissues were visualized using a Leica SP8 Confocal.

dHNF4 staining was conducted by dissecting larvae in 1x PBS and fixing for 30 min in 4% paraformaldehyde in 1x PBS at room temperature. Fixed tissues were then washed three times for 5 min each with PT (PBS with 0.1% Triton X-100) and blocked for 30 min in NGS-PT (PT with 5% NGS) at room temperature. Blocked tissues were incubated overnight at 4°C with a previously described Guinea Pig anti-HNF4 (kindly provided by by Gilles Storelli, (39)) diluted 1:100 in NGS-PT. Tissues were subsequently washed three times for 5 min each with PT and then incubated overnight with DyLight 488 Goat anti-Guinea Pig secondary antibody (#SA5-10094 Invitrogen) diluted 1:1000 in PT at 4°C. Tissues were then washed three times for 5 min each in PT and mounted in Vectashield with 4',6-diamidino-2-phenylindole (Vector Laboratories; H-1200-314 10). Stained tissues were visualized using a Leica SP8 Confocal.

### Fat body staining

Nile red staining was conducted on L2 fat bodies as previously described (45). Briefly, dissected tissues were fixed with 4% paraformaldehyde in PBS for 30 min, washed with PBS + 0.05% Tween 20 (PBST) three times for 5 min each, and then incubated for 1 h at room temperature in a 1:1000 dilution of Nile Red stock (10 mg/ml in acetone) in 1X PBS. Tissues were washed three times for 5 min each in PBST, mounted in Vectashield (Vector Laboratories), and imaged on a Leica SP8 Confocal at 568 nm.

Solvent Black 3 (SB3) staining was conducted as described (46, 47). Fat bodies were fixed with 4% formaldehyde, rinsed twice with PBS, once with 50% ethanol, and then stained for 2 min at room temperature using filtered 0.5% SB3 (CAS Number 4197-25-5; Sigma 199664) dissolved in 75% ethanol. Samples were sequentially rinsed with 50% ethanol, 25% ethanol, and PBS. Stained tissues were mounted on a microscope slide with Vectashield (Vector Laboratories; H-1200-10) and visualized using an EVOS FL Auto from Life Technologies. Using ImageJ, an region of interest selection was made and then inverted (48). The measure function was then used to quantify stain intensity over the area of the selection.

### TAG, trehalose, and protein measurements

TAG, trehalose, and protein were quantified from larval extracts using previously described protocols (detailed step-by-step instructions can be found in (47). For all three assays, 25 mid-L2 larvae of the appropriate genotypes were then collected in 1.5 ml microfuge tubes and washed three times with pH 7.0 (PBS).

Samples collected for TAG quantification were subsequently homogenized in 100 μl of cold PBST, 10 μl of homogenate were set aside to quantify soluble protein, and the remaining 90 μl were heat-treated for 10 min at 70°C. The resulting homogenate was assayed for TAGs by treating with TAG reagent (Sigma T2449) at 37°C for 30 min, centrifuged to pellet insoluble debris, and 20 μl of clear supernatant was transferred to a clear 96-well plate. One hundred microliters of free glycerol reagent (Sigma F6428) was added to each well and the entire plate was incubated at 37°C for 5 min. Absorbance was then measured at 540 nm using a plate reader.

Samples for trehalose assays were homogenized in 100 μl cold trehalase buffer (5 mM Tris pH 6.6, 137 mM NaCl, 2.7 mM KCl), 10 μl of homogenate were set aside to quantify soluble protein, and the remaining 90 μl were heat-treated for 10 min at 70°C. The homogenate was centrifuged to remove insoluble material, and 30 μl of the clear supernatant was transferred into a new 1.5 ml microfuge tube. Thirty microliters of trehalase buffer + porcine trehalase (3 μl of trehalase [Sigma T8778-1UN]/1 ml of trehalase buffer) were added to each tube and the reaction was placed in a 37°C incubator for 24 h. Following a brief centrifugation, 30 μl of treated homogenated were transferred into a clear bottom 96-well plate and 100 μl of glucose oxidase reagent (Sigma; GAGO-20) were added to each well. Following a 1 h incubation at 37°C, 100 μl of 12 N H_2_SO_4_ was added to each well to terminate the reaction. The plate was then analyzed in a plate reader to measure absorbance at 540 nm.

A Bradford assay was used to quantify soluble protein levels in the 10 μl aliquot of homogenate set aside prior to the 70°C heat treatment (49).

### HNF4 LBD assays

Analysis was conducted using either *w*^*1118*^*; hs-GAL4-dHNF4 UAS-nlacZ; dERR*^*1*^ mutants, *w*^*1118*^*; hs-GAL4-dHNF4 UAS-nlacZ; dERR*^*1/+*^ heterozygous controls, or *w*^*1118*^*; UAS-nlacZ* negative control larvae. Tissues from mid-L2 larvae were dissected and fixed as described (43). Briefly, molasses-yeast plates containing mid-L2 larvae were heat-treated for 30 min at 37°C and then allowed to recover for 2 h at 25°C. Larvae were dissected in 1x PBS, fixed for 20 min in 4% PFA in 1x PBS at room temperature, and stained in 0.2% X-Gal staining solution for 2 h at 37°C. Following the staining period, tissues were washed three times for 5 min each in 1x PBS, mounted in a solution of 50% glycerol, and imaged immediately following mounting using an EVOS FL Auto from Life Technologies.

Using ImageJ (v1.53t), the color channels were split, and the red channel was used to make an region of interest selection. This selection was inverted and then a threshold was manually determined to visualize the stained area within the selection (48). The staining was then quantified using the measure function.

### Lipidomic analysis

#### Chemicals

LC/MS-grade solvents and mobile phase modifiers were obtained from Honeywell Burdick & Jackson, Morristown, NJ (acetonitrile, isopropanol, formic acid), Thermo Fisher Scientific, Waltham, MA (methyl *tert*-butyl ether [MTBE]) and Sigma-Aldrich/Fluka, St. Louis, MO (ammonium formate, ammonium acetate). Lipid standards were obtained from Avanti Polar Lipids, Alabaster, AL (EquiSPLASH LIPIDOMIX (330731)) and Cayman Chemical, Ann Arbor, MI, (Palmitic acid d-31 (16497)).

#### Sample preparation

Extraction of lipids was carried out using a biphasic solvent system of cold methanol, MTBE, PBS, and water as previously described with some modifications (50). In a randomized sequence, to each sample was added 225 μl MeOH with internal standards and 188 μl PBS. Samples were homogenized for 30 s, transferred to 13 × 100 mm screw-capped glass test tubes containing 750 μl MTBE, and then incubated on ice with occasional vortexing for 1 h. Following incubation, samples were centrifuged at 15,000 *g* for 10 min at 4°C. The organic (upper) layer was collected, and the aqueous (lower) layer was re-extracted with 1 ml of 10:3:2.5 (*v/v/v*) MTBE/MeOH/dd-H2O, briefly vortexed, incubated at room temperature, and centrifuged at 15,000 *g* for 10 min at 4°C. Upper phases were combined and evaporated to dryness under nitrogen. Lipid extracts were reconstituted in 800 μl of 4:1:1 (v/v/v) isopropanol/acetonitrile/water and transferred to LC-MS vials for analysis. Concurrently, a process blank sample was prepared and pooled quality control (QC) samples were prepared by taking equal volumes from each sample after final resuspension.

#### Mass spectrometry analysis of samples

Lipid extracts were separated on an Acquity UPLC CSH C18 column (2.1 × 100 mm; 1.7 μm) coupled to an Acquity UPLC CSH C18 VanGuard precolumn (5 × 2.1 mm; 1.7 μm) (Waters, Milford, MA) maintained at 65°C connected to an Agilent HiP 1290 Sampler, Agilent 1290 Infinity pump, and Agilent 6545 Accurate Mass Q-TOF dual AJS-ESI mass spectrometer (Agilent Technologies, Santa Clara, CA). Samples were analyzed in a randomized order in both positive and negative ionization modes in separate experiments acquiring with the scan range *m/z* 100–1700. For positive mode, the source gas temperature was set to 225°C, with a drying gas flow of 11 l/min, nebulizer pressure of 40 psig, sheath gas temp of 350°C, and sheath gas flow of 11 l/min. VCap voltage is set at 3500 V, nozzle voltage 500 V, fragmentor at 110 V, skimmer at 85 V, and octopole radio frequency peak at 750 V. For negative mode, the source gas temperature was set to 300°C, with a drying gas flow of 11 l/min, a nebulizer pressure of 30 psig, sheath gas temp of 350°C, and sheath gas flow 11 l/min. VCap voltage was set at 3500 V, nozzle voltage 75 V, fragmentor at 175 V, skimmer at 75 V, and octopole RF peak at 750 V. Mobile phase A consisted of ACN:H_2_O (60:40, *v/v*) in 10 mM ammonium formate and 0.1% formic acid, and mobile phase B consisted of IPA:ACN:H_2_O (90:9:1, *v/v/v*) in 10 mM ammonium formate and 0.1% formic acid. For negative mode analysis the modifiers were changed to 10 mM ammonium acetate. The chromatography gradient for both positive and negative modes started at 15% mobile phase B then increased to 30% B over 2.4 min, it then increased to 48% B from 2.4 to 3.0 min, then increased to 82% B from 3 to 13.2 min, then increased to 99% B from 13.2 to 13.8 min where it was held until 16.7 min, then returned to the initial conditions and equilibrated for 5 min. Flow was 0.4 ml/min throughout, with injection volumes of 1 μl for positive and 10 μl negative mode. MS/MS was conducted using iterative exclusion, with the same LC gradient at collision energies of 20 V and 27.5 V in positive and negative modes, respectively.

#### Analysis of mass spectrometry data

For data processing, Agilent MassHunter (MH) Workstation and software packages MH qualitative and MH quantitative were used. The pooled QC (n = 8) and process blank (n = 4) were injected throughout the sample queue to ensure the reliability of acquired lipidomics data. For lipid annotation, accurate mass and MS/MS matching was used with the Agilent Lipid Annotator library and LipidMatch (51). Results from the positive and negative ionization modes from Lipid Annotator were merged based on the class of lipid identified. Data exported from MH quantitative were evaluated using Excel, where initial lipid targets are parsed based on the following criteria. Only lipids with relative standard deviations less than 30% in QC samples are used for data analysis. Additionally, only lipids with background area under the curve counts in process blanks that are less than 30% of QC are used for data analysis. The parsed excel data tables are normalized based on the ratio to class-specific internal standards, then to tissue mass prior to statistical analysis.

#### Statistical analysis and data visualization

Multivariate analysis was performed using MetaboAnalyst 6.0 (52). Statistical models were created for the normalized data after logarithmic transformation (base 10) and Pareto scaling. Initial pass for the volcano plot used a fold change (FC) cut off of 1.5, adjusted *P* value cut off: 0.05, and multiple testing correction:false discovery rate.

### Sample preparation and RNA extraction for RNA-Seq

*dERR*^*1/+*^*and dERR*^*1/2*^ L2 larvae (66–68 h) were raised at 25°C on molasses plates covered with yeast. Larval stage was confirmed by spiracle morphology prior to dissection. Larvae were washed and dissected in 1x PBS in groups of 5 to ensure speed of dissection and preservation of RNA quality. A total of 15 larvae were dissected to make three sets of technical replicates per genotype. No. 5 Biology Grade Dumont INOX dissecting forceps were used to decapitate larvae and dissect the fat body from surrounding tissue. Complete RNA was extracted using RNeasy Plus Micro Kit (74,034, Qiagen) following manufacturer’s instructions. The fat body was quickly moved to a 1.7 ml Eppendorf tube with a small volume (50 ml) of the RNA lysis tissue Plus Buffer with beta-mercaptoethanol added. The tissue was homogenized in the solution with a sterile pestle while keeping the tube on ice. The homogenized samples were flash-frozen in liquid nitrogen and stored at −80°C until further processing. After removal of samples from the −80°C, an additional 300 ul of RNA lysis tissue Plus was added to the sample just before it completely thawed. Then, 350 ul of 70% ethanol was added and the combined 700 ul pipetted forcefully to mix. Immediately thereafter, the sample was transferred to an RNeasy MinElute spin column before proceeding with the rest of the manufacturer protocol (starting at RNAeasy kit step 5).

### RNA-seq data processing

RNA-seq processing scripts are included in supplemental Fig. S3 and all commands are run with default settings unless otherwise stated. FastQC (0.12.1) was used to assess read quality prior to further processing (http://www.bioinformatics.babraham.ac.uk/projects/fastqc). The D. melanogaster BDGP6.46 hard-masked assembly (53) and v110 annotation (54) were downloaded from ENSEMBL, and malformed entries in the annotation file were corrected using AGAT (1.2.0) ‘agat convert sp gxf2gxf.pl’ (53). gffread (0.12.7) was used to create a transcript sequence file (54). The genome assembly was concatenated, followed by decoy-aware indexing with Salmon (1.10.2) (55). Salmon quantification was then performed with nondefault settings: ‘--seqBias --gcBias --posBias --softclip softclipOverhangs --numGibbsSamples 100’.

### RNA-seq downstream analysis

The downstream RNA-seq analysis scripts are included in supplemental Fig. S2 and all analyses were performed with R (4.3.2). Salmon transcript abundance estimates were aggregated to gene level and imported into R using tximport (1.30.0) (56). For QC, PCAtools (2.14.0, https://github.com/kevinblighe/PCAtools was utilized for principal component analysis, using DESeq2 (1.42.0, (57) `rlog()’-normalized counts with the design “∼ condition + source + condition:source’ and blind = FALSE, where “condition” is the ERR mutation status and “source” the tissue source type (i.e., whole body or fat body). For differential expression, genes were considered to have a significant interaction term, that is, their log2FC is significantly different depending on whether the tissue was from the whole body or fat body, if the false discovery rate <0.05. Separate contrasts were performed with a simplified design (“∼ condition_source” where “condition_source” is the concatenated “condition” and “source” factor levels) to compare whole body or fat body ERR mutant versus controls, and apeglm (1.24.0, (58) was used for log FC shrinkage as well as testing against |log2FC| > 1, with significant genes having an s-value < 0.005. An additional analysis was performed where only genes that were both differentially expressed (s-value < 0.005) and with a significant interaction term (false discovery rate < 0.05) were considered. Volcano and bar plots were generated with ggplot2 (3.4.4), euler plots with eulerr (7.0.0, (59), and heat maps with ComplexHeatmap (2.18.0, (60). The `fora()’ function from fgsea (1.28.0, (61) was used to perform hypergeometric enrichment analysis using the following gene sets pulled from Molecular Signatures Database (MSigDB, (62) via the msigdbr (7.5.1, (63) library: hallmark gene sets, chemical and genetic perturbations, reactome pathways, gene ontology (GO) molecular function and biological process, and human phenotype ontology. Enrichment plots were generated using ggplot2.

### GO analysis using PANGEA

RNAseq data were analyzed using PANGEA (64). Genes that were significantly downregulated or upregulated were analyzed for GO Enrichment using the SLIM2 GO BP and FlyBase signaling pathway (experimental evidence) sets (65).

## Results

### Lipidomic analysis of *dERR* mutants reveals significant reduction of TAG levels

**Fig. 1:**
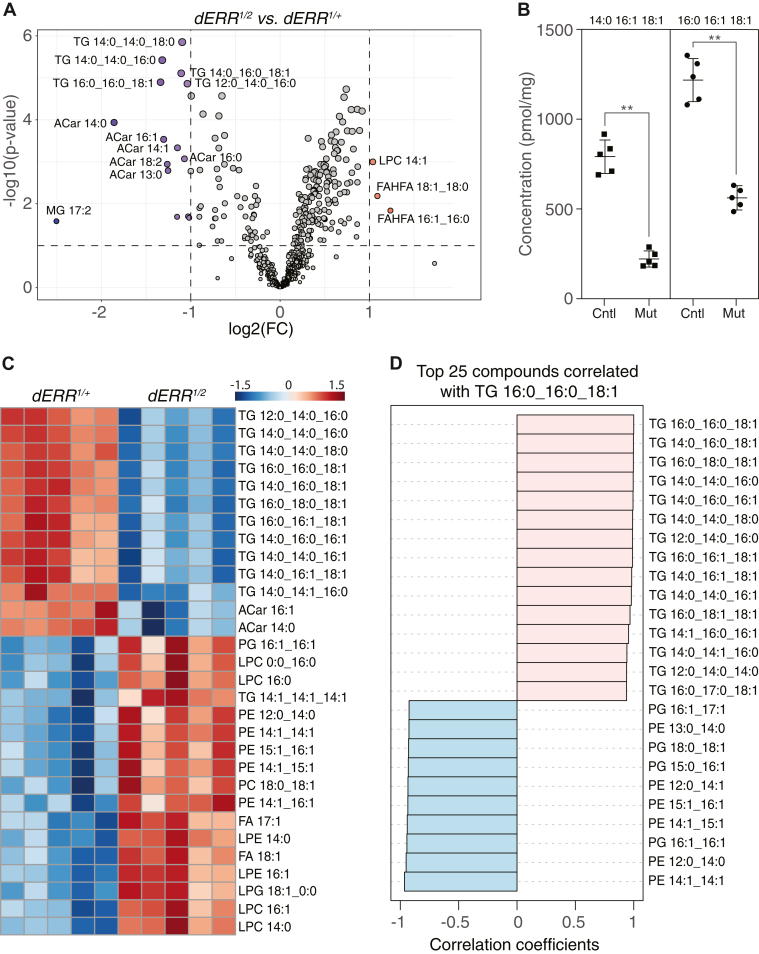
Quantitative lipidomic analysis of *dERR* mutant larvae. *dERR*^*1/2*^ mutant larvae and *dERR*^*1/+*^ controls were analyzed using a quantitative lipidomic approach that measured the concentration of 537 lipid species. A: Differences in lipid abundance between the mutant and control groups are represented with a volcano plot. Dashed vertical line represents a log2 fold change (FC) of 2, and dashed horizontal line represents *P* = 0.1. B: Abundance of the triglycerides 16:0_16:1_18:1 and 14:0_16:1_18:1 in *dERR* mutant larvae (Mut) as compared to controls (Cntl). ∗∗*P* < 0.01. *P-*value calculated using a Kolmogorov-Smirnov test. All panels were generated using Metaboanalyst 6.0. C: The top 30 significantly altered lipid species between mutant and control larvae are represented in a heat map and ordered based on *P* value calculated using a two sample *t* test. D: The top 25 compounds correlated with TAG 16:0_16:0_18:1. n = 5 biological replicates for each genotype and each replicate consists of 20 mid-L2 larvae.

To determine how dERR promotes larval lipid accumulation, we first used a quantitative lipidomics approach to measure the concentration of 537 lipid species in both *dERR*^*1/2*^ mutants and *dERR*^*1/+*^ heterozygous controls (supplemental Table S1). Principal component and differential expression analyses of the resulting datasets revealed significant differences between control and mutant larvae (supplemental Fig. S1A), with a total of 17 lipid species significantly changing in abundance (log2 FC ≥ 1; adjusted *P* < 0.05; Fig. 1A, supplemental Table S2). Among these lipids, the most significantly altered species are TAGs (Fig. 1A, B), with six individual TAG molecules meeting or exceeding the cutoff threshold. Five of these six lipids rank among the most abundant TAG species present within larvae (Table 1), and subsequent analyses revealed that nine of the 25 most abundant TAGs are significantly decreased in *dERR* mutants relative to heterozygous controls. Notably, the two TAGs with the highest concentration in control larvae, TG_16:0_16:1_18:1 and TG_14:0_16:1_18:1, are decreased ∼40% in mutant larvae relative to the controls (Table 1, Fig. 1B, C; q < 0.01). Consistent with these findings, we observed similar changes using a biochemical assay, which revealed that *dERR*^*1/2*^ and *dERR*^*1*^*/Df* mutant larvae exhibit a 50–60% decrease in TAG levels when compared with *dERR*^*1/+*^ heterozygous control larvae (supplemental Fig. S2). We would also note that the decreased TAG levels measured in both the lipidomic and biochemical analyses are comparable to the decreases observed in previous studies (35). Together, our findings confirm that loss of dERR activity results in a significant decrease of TAG stores.

**Table 1:** Quantification of the 25 most abundant TAG species in *dERR^1/2^* mutants and *dERR^1/+^* controls.

TAG Species	Control (pmol/mg; n = 6)	SD	Mutant (pmol/mg; n = 5)	SD	Mutant/Control	q value
**TG 16:0_16:1_18:1**	**1276.17**	**153.86**	**766.45**	**80.51**	**0.60**	**0.003**
**TG 14:0_16:1_18:1**	**1271.18**	**167.92**	**791.70**	**93.22**	**0.62**	**0.004**
TG 14:1_16:0_16:1	903.09	153.44	615.62	75.85	0.68	0.017
**TG 14:0_16:0_18:1**	**856.73**	**138.95**	**375.05**	**52.73**	**0.44**	**0.003**
TG 16:1_16:1_18:1	754.70	122.51	584.03	59.68	0.77	0.047
TG 16:1_16:1_16:1	749.06	154.24	617.12	81.21	0.82	0.162
**TG 14:0_16:0_16:1**	**626.57**	**125.78**	**319.09**	**46.52**	**0.51**	**0.007**
**TG 16:0_16:0_18:1**	**611.16**	**132.51**	**222.00**	**44.85**	**0.36**	**0.004**
TG 14:0_14:1_18:1	583.09	89.84	462.89	38.80	0.79	0.049
TG 14:0_14:0_16:1	527.24	109.46	293.65	43.97	0.56	0.012
TG 16:0_18:1_18:1	521.43	108.87	312.75	53.73	0.60	0.016
TG 14:0_14:1_16:1	470.38	68.11	419.75	37.19	0.89	0.204
TG 14:1_16:0_18:1	432.21	62.46	338.10	23.23	0.78	0.035
TG 14:1_16:1_16:1	377.32	58.61	415.20	42.87	1.10	0.275
TG 14:1_16:1_18:1	360.67	73.12	329.20	16.81	0.91	0.369
TG 12:0_14:0_16:1	347.04	59.99	261.09	41.79	0.75	0.049
TG 16:1_18:1_18:1	346.07	50.81	273.06	23.59	0.79	0.042
TG 14:0_14:0_14:1	304.94	42.90	239.22	20.98	0.78	0.035
TG 12:0_14:1_16:1	297.68	27.90	325.17	38.30	1.09	0.264
**TG 12:0_14:0_16:0**	**242.52**	**58.48**	**106.91**	**15.77**	**0.44**	**0.011**
**TG 16:0_18:0_18:1**	**239.02**	**44.79**	**111.52**	**18.69**	**0.47**	**0.004**
TG 14:0_14:1_14:1	223.19	26.35	248.52	18.69	1.11	0.153
**TG 14:0_14:1_16:0**	**221.80**	**32.39**	**133.63**	**12.98**	**0.60**	**0.005**
TG 14:1_14:1_16:1	182.39	24.52	227.03	19.65	1.24	0.029
**TG 14:0_14:0_16:0**	**165.38**	**31.38**	**61.49**	**10.20**	**0.37**	**0.004**

q < 0.01 for rows highlighted in bold.

TAG species ordered according to abundance.

ERR, estrogen-related receptor; SD, standard deviation; TAG, triglyceride.

Beyond the observed changes in TAG abundance, the levels of several other lipids are significantly altered in *dERR* mutants when compared with controls. Notably, acylcarnitine (ACAR) species were downregulated > 2-fold in mutants when compared with the heterozygous control (supplemental Table S2, Fig. 1A). Since ACARs serve as a key intermediate during the transport of long-chain fatty acids into the mitochondria, our findings raise the possibility that fatty acid β-oxidation is decreased in *dERR* mutants. Moreover, when we reanalyzed the lipidomic data using a less stringent cutoff (absolute FC > 1.5; adjusted *P* < 0.05; supplemental Table S3), several phospholipids (phosphatidylcholine; phosphatidylethanolamine; phosphatidylglycerols); and lysophospholipids (lysophosphatidylcholine and lysophosphatidylethanolamine) were found to be elevated in *dERR* mutants (Fig. 1C). In fact, a correlation analysis of TG_16:0_16:1_18:1 revealed a significant inverse correlation between phosphatidylethanolamine levels and TAG levels in *dERR* mutant larvae (Fig. 1D). Overall, our analysis reveals that of those lipids significantly altered in *dERR* mutants, there are significant decreases in stored lipids and acylcarnitines as well as a subtle increase in phospholipids.

### dERR expression within the fat body promotes TAG accumulation

**Fig. 2:**
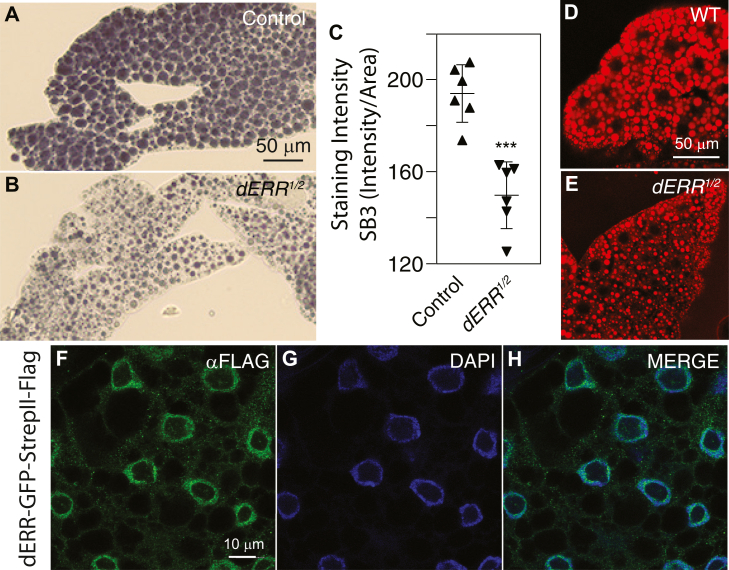
*dERR* mutants exhibit decreased TAG accumulation in the larval fat body. A–D: Fat bodies from (A) *dERR*^*1/+*^ heterozygous controls and (B) *dERR*^*1/2*^ mutants were dissected from mid-L2 larvae (∼60 h after egg-laying), fixed, and stained with Solvent Black 3 (SB3). C: SB3 staining intensity relative to area was quantified for fat bodies from six control and mutant larvae. D and E: Fat bodies from (D) *dERR*^*1/+*^ heterozygous controls and (E) *dERR*^*1/2*^ mutants were dissected from mid-L2 larvae fixed, and stained with Nile red. Scale bar in (A) also applies to (B). Scale bar in (D) also applies to (E). F–H: Fat bodies from mid-L2 larvae expressing a previously described dERR-GFP-StrepII-Flag transgene were stained with αFlag antibody and DAPI. Scale bar in panel (F) applies to (G) and (H). Data analyzed using an ordinary ANOVA test followed by a Tukey’s multiple comparison test. ∗∗∗*P* < 0.001. DAPI, 4',6-diamidino-2-phenylindole; TAG, triglyceride.

Previous studies that examined the influence of dERR on larval TAG metabolism relied on measurements from whole animal homogenates (35). To better understand the tissue-specific mechanisms by which ERR regulates TAG accumulation, we focused on the fat body, which is the primary TAG storage site (66, 67). As an initial approach to determine whether the global decrease in *dERR* mutant TAG levels was reflected in the fat body, we stained mutant and control larvae using the lipophilic dye SB3. Consistent with the whole-body TAG measurements (Fig. 1 and supplemental Fig. S2), SB3 staining was significantly reduced in *dERR*^*1/2*^ mutant fat body when compared with *dERR*^*1/+*^ heterozygous controls (Fig. 2A–C). Similarly, *dERR* mutant fat bodies exhibited lower levels of Nile red staining (Fig. 2D, E), thus providing further evidence that loss of dERR activity results in decreased fat body TAG accumulation.

The SB3 and Nile red staining results raised the question as to whether dERR can function autonomously within fat body cells to promote TAG accumulation. To test this possibility, we first examined if dERR is expressed in L2 larval fat bodies using a previously described transgene that expresses a *dERR-FLAG-GFP* fusion protein from the endogenous *dERR* promoter (42). Immunohistochemistry using an anti-FLAG antibody revealed that dERR is highly expressed within larval fat bodies and primarily localized to the nucleus, although we also noted low levels of dERR present within the cytosol (Fig. 2F–H). In contrast, we observed no staining in a control strain lacking the transgene (supplemental Fig. S3). This result, together with previous observations demonstrating that a dERR LBD reporter is active within larval fat body cells (43), suggested that *dERR* mutants exhibit decreased TAG levels due, in part, to loss of dERR expression within this tissue.

**Fig. 3:**
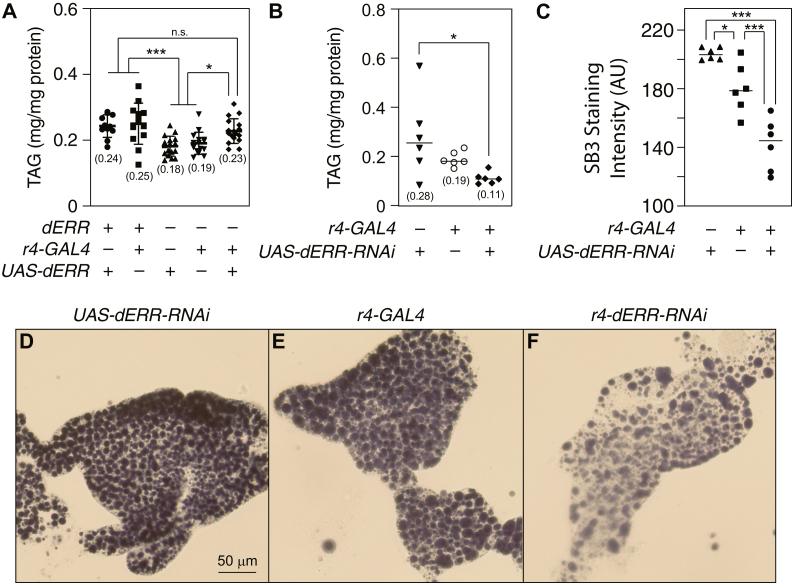
dERR promotes TAG accumulation in the larval fat body. A: TAG levels were quantified relative to soluble protein in whole-body extracts from the heterozygous control genotypes *r4-Gal4/+*; *dERR*^*2/+*^ and *UAS-ERR/+*; *dERR*^*1/+*^, the *dERR* mutant control genotypes *r4-Gal4/+*; *dERR*^*1/2*^ and *UAS-ERR/+*; *dERR*^*1/2*^, and mutant larvae expressing the rescuing transgene in the fat body (*r4-Gal4 +/+ UAS-dERR*; *dERR*^*1/2*^). B: TAG levels were quantified relative to soluble protein in whole-body extracts from heterozygous control strains *r4-Gal4/+* and *UAS-dERR-RNAi/+*, as well as larvae expressing the *UAS-dERR-RNAi* transgene under the control of *r4-Gal4* (*r4-dERR-RNAi*). C–F: SB3 staining was used to measure TAG levels in control larvae (*UAS-ERR-RNAi/+* and *r4-Gal4/+*) as well as in *r4-dERR-RNAi* larvae. C: Quantification of SB3 staining intensity in control and *r4-dERR-RNAi* larval fat bodies. D–F: Representative images of SB3 staining for each genotype. Data were analyzed using an ordinary ANOVA test followed by a Tukey’s multiple comparison test. ∗*P* < 0.05; ∗∗*P* < 0.01; and ∗∗∗*P* < 0.001. SB3, Solvent Black 3; TAG, triglyceride.

To test the hypothesis that dERR functions within the larval fat body to drive TAG accumulation, we used the fat body–specific driver *r4-Gal4* to (i) express a *UAS-dERR* transgene in a *dERR* mutant background, and (ii) express a *UAS-ERR-RNAi* transgene in the wild-type fat body. Consistent with our model, expression of dERR only in fat body cells is sufficient to significantly increase TAG levels in *dERR* mutants as determined by whole-body TAG measurements (Fig. 3A), as well as SB3 staining of isolated fat bodies (supplemental Fig. S4). Similarly, we observed a decrease in the TAG levels of *r4-ERR-RNAi* larvae when compared with the control strains (Fig. 3B). In this regard, we would note that whole animal TAG levels were highly variable in the *UAS-ERR-RNAi* control strain when compared with *r4-Gal4* control larvae and *r4-ERR-RNAi* larvae. As a result, the differences in TAG abundance between the *r4-Gal4* controls and *r4-ERR-RNAi* samples failed to reach a significance threshold of *P* < 0.05 in an ANOVA followed by Tukey’s multiple comparison test (Fig. 3B). We would note, however, that the decrease in TAG levels between the *r4-Gal4* controls and *r4-ERR-RNAi* samples were nearly identical to those observed in *dERR*^*1/2*^ and *dERR*^*1*^*/Df* mutants when compared to *dERR^1/+^* heterozygous controls (supplemental Fig. S2). Also, a pairwise statistical analysis between only these two sample groups using a two-tailed Mann–Whitney test revealed a *P* value of <0.01. Moreover, when we stained *r4-ERR-RNAi* larvae and the two control strains using SB3, *r4-ERR-RNAi* fat bodies displayed a consistent and significant decrease in TAG when compared with either control genotype (Fig. 3C–F). Together, these observations support a model in which dERR acts within larval fat body cells to promote normal TAG homeostasis.

### Genes involved in carbohydrate metabolism and β-oxidation exhibit decreased expression in *dERR* mutant fat bodies

**Fig. 4:**
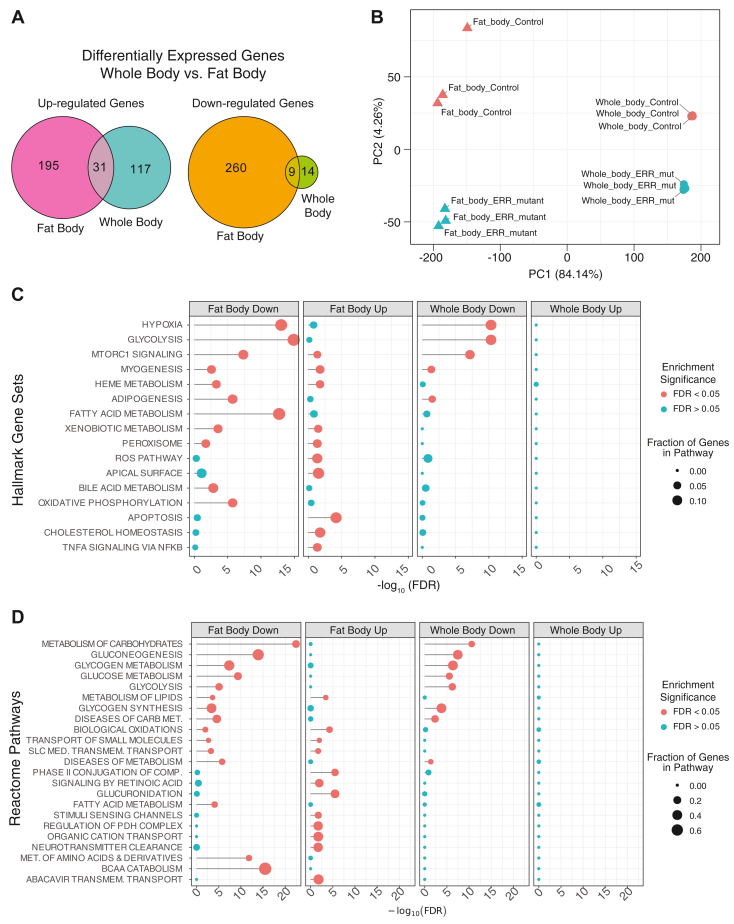
Tissue-specific RNA-seq analysis of *dERR* mutants compared with heterozygous controls. RNA isolated from whole mid-L2 *dERR*^*1/2*^ mutant larvae and *dERR*^*1/+*^ controls, as well as from dissected fat bodies from those two genotypes, were compared using RNA-seq. A: Euler plot indicating the number of genes that were either downregulated or upregulated in both *dERR* mutant fat bodies and whole animals. B: principal component analysis plot of the RNA-seq samples. Samples separate along the principal component 1 axis based on tissue and the principal component 2 axis according to genotype. C and D: Term enrichment analysis of the RNA-seq data were conducted using the (C) “Hallmark Gene Sets” and (D) “Reactome Pathways” collections in the molecular signatures database (MSigDB). Data from the whole body and fat body were analyzed separately as labeled.

To better understand the underlying metabolic mechanisms leading to decreased TAG levels within *dERR* mutant fat bodies, we used RNA-seq to analyze gene expression in (i) the fat bodies dissected from *dERR*^*1/2*^ mutants as well as *dERR*^*1/+*^ heterozygous controls and (ii) whole larvae of the same genotypes (supplemental Tables S4, S5). These analyses revealed gene expression changes within the fat body that were unique compared to those observed in the whole animal analysis; only 40 genes exhibited significantly altered expression levels in both datasets (Fig. 4A, supplemental Tables S6 and S7), with most of these genes encoding enzymes involved in glycolysis and carbohydrate metabolism (supplemental Tables S6). In addition, principal component analysis highlighted that the tissue type segregated along the principal component 1, which accounts for over 84% of the variance (Fig. 4B), while genotype separated along principal component 2 axis and accounted for less than 5% of the variance (Fig. 4B). This supports the conclusion that the *dERR* mutant gene expression profile within the fat body significantly differs from that of the whole animal.

To better understand how dERR influences gene expression, we conducted a series of GO analyses of the whole body and fat body RNA-seq datasets. Using the Hallmark Gene Sets and Reactome Pathway databases, GO analysis revealed whole body enrichment for genes associated with metabolic processes, with the most significantly overrepresented GO categories associated with carbohydrate metabolism (e.g., hypoxia, glycolysis, metabolism of carbohydrates) among the downregulated genes (Fig. 4C, D, third panel). In the fat body dataset, however, there is additionally significant enrichment for fatty acid metabolism, adipogenesis (Fig. 4C), and metabolism of lipids (Fig. 4D). PANGEA analysis revealed a similar enrichment for GO categories associated with metabolism, with the RNA-seq data generated from the *dERR* mutant fat body exhibiting significant enrichments for genes involved in both carbohydrate and lipid metabolic processes (supplemental Tables S8, S9).

**Fig. 5:**
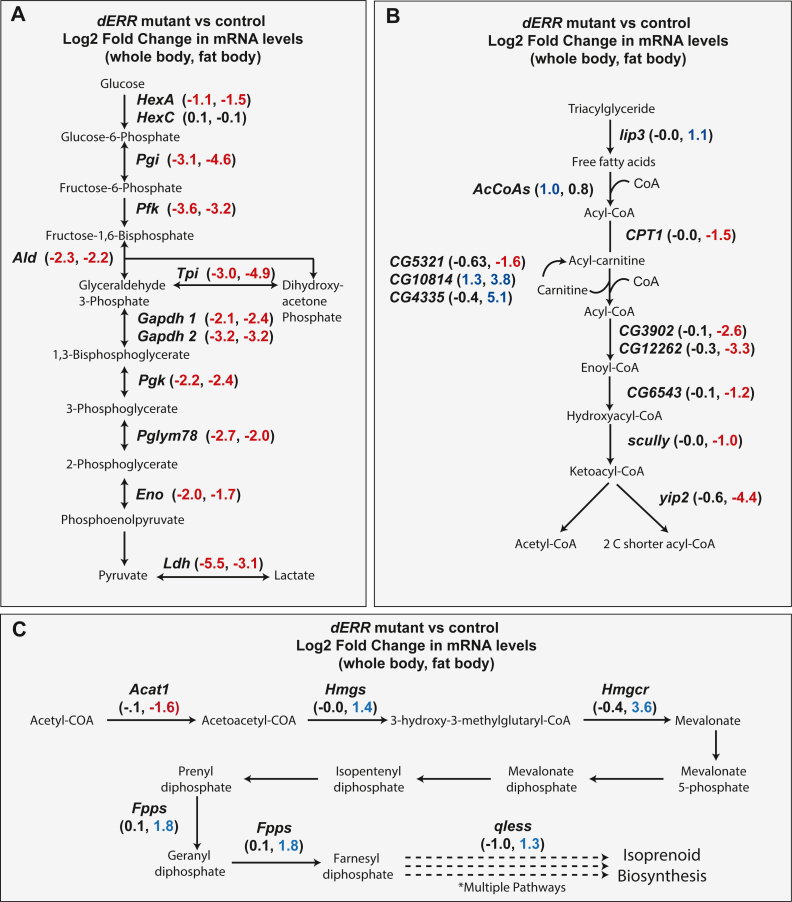
Expression of enzymes in glycolysis and β-oxidation are reduced in *dERR* mutants. Schematic diagrams illustrating how loss of dERR activity alters the expression of genes involved in (A) glycolysis, (B) β-oxidation, and (C) isoprenoid metabolism. Changes in gene expression are listed at each enzymatic step, with the first number in parenthesis indicating the log2 fold change in whole animal extract and the second number indicating the log2 fold change in only larval fat body. Log2 ≥1 was represented in blue and log2 ≤1 was represented in red for all panels.

A closer examination of the GO categories that are significantly enriched in *dERR* mutant fat bodies revealed disruption of three metabolic pathways. As expected, genes encoding every enzymatic step in glycolysis were significantly downregulated (Fig. 5A; supplemental Tables S4, S5). However, we also unexpectedly observed decreased expression of genes associated with fatty acid β-oxidation (Fig. 5B; supplemental Tables S4, S5), including the rate-limiting transporter *CPT1* (*whd*, FBgn0261862) and five genes that encode enzymes involved in the four downstream catabolic reactions (Fig. 5B; supplemental Tables S4, S5). None of these genes, however, exhibited significant expression changes in the *dERR* mutant whole animal RNA-seq dataset. We also noted that three genes associated with isoprenoid synthesis (*Hmgs*, *Hmgcr*, and *Fpps*) exhibited increased expression in *dERR* mutant fat bodies but were unchanged when measured in whole animal extracts (Fig. 5C; supplemental Tables S4, S5). Moreover, expression of a fourth gene involved in isoprenoid metabolism, *qless*, displayed opposite changes in the two datasets, with *qless* expression being increased *dERR* mutant fat bodies and decreased in the whole-body data (Fig. 5C; supplemental Tables S4, S5). Overall, these results suggest that dERR serves previously undescribed functions to influence fatty acid catabolism and isoprenoid synthesis within the larval fat body.

Our RNA-seq results suggest that the dERR gene expression program within the larval fat body could play a key role in coordinating carbohydrate availability with TAG storage. Notably, the observed gene expression profile is consistent with two previous observations: (i) although a high sugar diet results in elevated TAG accumulation in adult control animals, a high sugar diet did not result in increased TAG levels within *dERR* mutants (36). (ii) Expression of dERR solely within the larval fat body of *dERR* mutants reduces systemic levels of the disaccharide trehalose, the primary circulating sugar found within larval hemolymph (35, 47). Considering our RNA-seq findings, we reproduced the tissue-specific rescue experiment and confirmed that expressing *UAS-dERR* in the fat body of *dERR* mutants using an *r4-Gal4* transgene was sufficient to rescue trehalose levels (supplemental Fig. S5), again indicating that dERR activity within larval fat is sufficient to maintain glycemic control in the whole animal.

### HNF4 activity is decreased in *dERR* mutant fat bodies

Our RNA-seq analysis indicates that dERR can influence the expression of genes associated with fatty acid β-oxidation. Considering that all of the β-oxidation–related genes downregulated in *dERR* mutants are known targets of HNF4 (39), our findings raise the possibility that dHNF4 activity is altered in *dERR* mutant fat bodies. As a first step toward analyzing this possibility, we first examined dHNF4 expression in *dERR* mutants. We found no evidence, however, that dHNF4 expression was altered in *dERR* mutants, as *dHNF4* transcript abundance was unchanged in the RNA-seq analysis, (log2 FC in *dERR* mutants versus controls = 0.006; supplemental Table S4) and dHNF4 protein levels appear unchanged in *dERR* mutant fat bodies as compared with controls (supplemental Fig. S6). We also compared preexisting dERR and dHNF4 chromatin immunoprecipitation sequencing datasets (supplemental Table S11, (36, 68)), with the goal of determining if these NRs coregulate target genes. Our analysis, however, revealed little overlap (supplemental Table S11), with only four genes being bound by both dERR and dHNF4, none of which were differentially expressed in *dERR* mutant fat bodies.

**Fig. 6:**
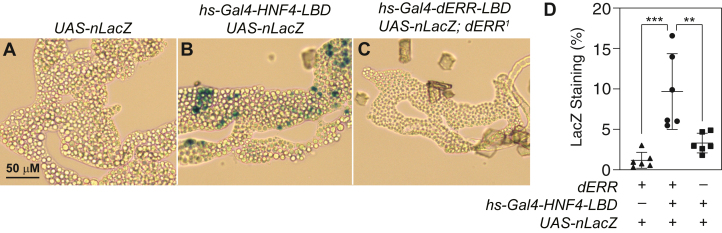
dHNF4 activity reporter in a *dERR* mutant background. HNF4 activity was visualized via X-Gal staining using *hs-Gal4-dHNF4-LBD* and *UAS-nLacZ* transgenes that were placed in a *dERR* mutant genetic background. Figure shows staining for (A) *UAS-nLacZ* control strain, (B) *hs-Gal4-dHNF4-LBD, UAS-nLacZ; dERR*^*1/+*^ heterozygous control, and (C) *hs-Gal4-dHNF4-LBD, UAS-nLacZ; dERR*^*1*^ mutants. D: X-Gal staining intensity relative to area was quantified for each genotype in (A–C). Data were analyzed using an ordinary ANOVA test followed by a Tukey’s multiple comparison test. ∗∗*P* < 0.01 and ∗∗∗*P* < 0.001. LBD, ligand-binding domain.

As an alternative approach towards examining the putative relationship between dERR and dHNF4, we used a dHNF4-LBD reporter (*hs-Gal4-HNF4-LBD, UAS-nLacZ*) to examine dHNF4 transcriptional activity within *dERR* mutants. This HNF4-LBD reporter has previously been shown to be active at low levels in the larval fat body under fed conditions (39). Consistent with these prior studies, the HNF4-LBD reporter was active at detectable but low levels in subsets of fat body cells in *dERR*^*1/+*^ heterozygous controls (Fig. 6B, D), however, reporter activity was nearly undetectable in the *dERR*^*1/2*^ mutant background (Fig. 6C, D). In fact, the amount of lacZ staining in *hs-Gal4-HNF4-LBD UAS-nLacZ; dERR*^*1/2*^ larvae (Fig. 6C, D) was often indistinguishable from the *UAS-nLacZ* control strain (Fig. 6A), thus indicating that HNF4 activity is reduced in *dERR* mutants. Considering that the *hs-Gal4-HNF4-LBD UAS-nLacZ* reporter system does not include a dERR binding site, our observations suggest that changes in dHNF4 activity within *dERR* mutants is the indirect effect of changes in either a key metabolite or dHNF4 cofactor. Regardless of the mechanism, our findings suggest that future studies should explore the interaction between these two NRs.

## Discussion

While loss-of-function mutations in both mouse *Esrra* and insect *ERR* genes are well-documented to induce a lean phenotype (34, 36, 37), the mechanisms by which these ERR family members function in adipose tissue to regulate TAG storage remain poorly understood. Here, we addressed this knowledge gap by conducting RNA-seq analysis of fat bodies isolated from *D. melanogaster ERR* mutants. Our studies revealed that fat bodies from *dERR* mutants exhibit significant decreases in the expression of genes involved in both carbohydrate and lipid catabolism. This finding was unexpected, as previous studies of dERR in both larvae and adults failed to identify a link between dERR activity and the expression of enzymes involved in lipid catabolism (35, 36). Considering that our lipidomic studies revealed significant decreases in both TAG and ACAR concentrations, our findings suggest that dERR can influence the expression of enzymes involved in lipid metabolism.

Our results raise the question as to how dERR regulates lipid metabolism in the fat body. While previous chromatin immunoprecipitation sequencing analysis of adult flies failed to identify direct binding of dERR to any genes involved in lipid synthesis or β-oxidation (36), this analysis relied on chromatin isolated from whole animals and would likely overlook fat body–specific dERR target genes. Consistent with this possibility, a recent study in *A. aegypti* demonstrated that mosquito ERR can bind to the promoter region of fatty acid synthase (34), thus suggesting that insect ERRs can directly regulate some genes involved in lipid metabolism. Future studies in *D. melanogaster* should focus on identifying the dERR target genes specifically within the fat body to determine what aspects of lipid metabolism are directly regulated by dERR activity.

Our RNA-seq analysis also unexpectedly revealed increased expression of enzymes involved in isoprenoid metabolism within *dERR* mutant fat bodies. Previous studies in *Drosophila* have implicated these enzymes in germ cell migration, juvenile hormone production, heart development, and signal transduction (69, 70, 71, 72, 73, 74, 75). While we are unsure as to why isoprenoid metabolism appears elevated in *dERR* mutants, we would note that *Drosophila* hedgehog signaling is dependent on this pathway (31, 74, 75). Since elevated hedgehog signaling is associated with decreased TAG accumulation (76, 77), perhaps increased expression of these enzymes is related to a metabolic feedback mechanism involving hedgehog signaling. Alternatively, isoprenoid metabolism also produces coenzyme Q_10_ (also known as ubiquinone) and the *Drosophila* gene *qless* is essential for Q_10_ synthesis (78). The observed increase of *qless* expression in *dERR* mutant fat bodies could therefore be related to production of a molecule in mitochondrial metabolism.

Beyond the potential that dERR acts within the fat body to directly regulate genes involved in fatty acid and isoprenoid metabolism, our analysis also suggests that changes in dHNF4 activity contribute to *dERR* mutant phenotypes. Not only does dHNF4 regulate expression of many of the β-oxidation genes that are downregulated in *dERR* mutant fat bodies (39) but activity of the HNF4-LBD reporter is also decreased in the fat body of *dERR* mutants. Moreover, mouse HNF4α is required for hepatic ACAR synthesis (79), a finding consistent with our lipidomic results which reveal decreased abundance of ACARs in *dERR* mutants. This putative interaction between dERR and dHNF4 raises questions as to how these two factors coordinate lipid metabolism with larval growth and development. One possibility is that these two NRs coregulate target genes, as ERRα and HNF4α binding sites were found to be enriched near a common set of genes in mouse liver (80), suggesting that dERR and dHNF4 might similarly bind to similar genomic regions within the larval fat body. However, we would note that the HNF4-LBD reporter assay described above relies on the Gal4/UAS system and thus should be insensitive to dERR-dependent effects on promoter binding, suggesting that an indirect interaction is most likely responsible for our observations.

A second potential mechanism linking dERR and dHNF4 transcriptional activity stems from the inability of *dERR* mutants to properly metabolize carbohydrates. The dHNF4 LBD is activated by free fatty acids (39), and since carbon flux from sugars to fatty acid synthesis is likely disrupted in *dERR* mutants, decreased free fatty acid abundance in *dERR* mutants could result in decreased dHNF4 activity. We would note, however, that free fatty acids are not decreased in the *dERR* mutants, thus if this mechanism is responsible for the change in dHNF4 activity, it would require fat body–specific depletion of fatty acids.

While the mechanism linking loss of dERR with apparent changes in dHNF4 activity will become the subject of future studies, these observations raise an exciting possibility. dHNF4 regulates both lipid and carbohydrate metabolism (39, 81), with previous studies highlighting the role of dHNF4 in controlling glucose-stimulated insulin secretion (68). Our studies reiterate that dERR activity within the fat body is sufficient to promote both TAG accumulation and maintain systemic trehalose levels. Future studies should therefore evaluate the possibilities that these NRs coordinately balance carbohydrate metabolism with TAG storage with a focus on potential links with endocrine signaling.

Finally, our study highlights the importance of using the fly to study the ERR family of NRs. As noted above, mice lacking ERRα are viable but lean. In contrast, *dERR* mutants are homozygous lethal, dying during the late L2 stage, raising questions as to why the fly mutant exhibits a more severe phenotype than the ERRα single mutant. This discrepancy likely reflects the complex interaction that exists between the mammalian ERR orthologs, as mammalian genomes encode three ERR homologs (ERRα, ERRβ, and ERRγ). While each mammalian ERR has specialized function (82), ERRα and ERRγ also have overlapping functions, as simultaneous loss of both ERRα and ERRγ in the heart, skeletal muscle, and brown fat result in metabolic phenotypes that are more severe than either single mutant (83, 84, 85, 86). Since flies encode a single ERR homolog, *dERR* genetic mutations represent the most severe loss of ERR activity in any system, as all other insect studies rely on RNAi-based approaches. Our analysis of dERR in the *Drosophila* fat body is therefore of importance as it provides an in depth analysis of how an ERR family member influences lipid storage in adipose tissue and suggests that additional studies of dERR can provide further insight toward the role of this ancient NR family in regulating lipid homeostasis.

## Data availability

Processed RNA-seq data are presented in supplemental Tables S4 and S5. Lipidomics data are available in supplemental Table S1. Original data are available in NCBI Gene Expression Omnibus (GSE 273774).

## Supplemental data

This article contains supplemental data (58, 60).

## Conflict of interest

The authors declare that they have no conflicts of interest with the contents of this article.

## References

[1] Garfinkel A.M., Ilker E., Miyazawa H., Schmeisser K., Tennessen J.M. (2024). Historic obstacles and emerging opportunities in the field of developmental metabolism - lessons from Heidelberg. Development.

[2] Miyazawa H., Aulehla A. (2018). Revisiting the role of metabolism during development. Development.

[3] Palm W., Rodenfels J. (2020). Understanding the role of lipids and lipoproteins in development. Development.

[4] Zhu H., Han M. (2014). Exploring developmental and physiological functions of fatty acid and lipid variants through worm and fly genetics. Annu. Rev. Genet..

[5] Watts J.L., Ristow M. (2017). Lipid and carbohydrate metabolism in caenorhabditis elegans. Genetics.

[6] Ho S.-Y., Thorpe J.L., Deng Y., Santana E., Derose R.A., Farber S.A. (2004). Lipid metabolism in zebrafish. Methods Cell Biol..

[7] Srinivasan S. (2015). Regulation of body fat in caenorhabditis elegans. Annu. Rev. Physiol..

[8] Baker E.R. (1985). Body weight and the initiation of puberty. Clin. Obstet. Gynecol..

[9] Huang L., Hou J.W., Fan H.Y., Tsai M.C., Yang C., Hsu J.B. (2023). Critical body fat percentage required for puberty onset: the Taiwan pubertal longitudinal study. J. Endocrinol. Invest..

[10] Li Y., Ma T., Ma Y., Gao D., Chen L., Chen M. (2022). Adiposity status, trajectories, and earlier puberty onset: results from a longitudinal cohort study. J. Clin. Endocrinol. Metab..

[11] Scheidl T.B., Brightwell A.L., Easson S.H., Thompson J.A. (2023). Maternal obesity and programming of metabolic syndrome in the offspring: searching for mechanisms in the adipocyte progenitor pool. BMC Med..

[12] Barrea L., Vetrani C., Verde L., Frias-Toral E., Garcia-Velasquez E., Ranasinghe P. (2022). Gestational obesity: an unconventional endocrine disruptor for the fetus. Biochem. Pharmacol..

[13] Marcus C., Danielsson P., Hagman E. (2022). Pediatric obesity-Long-term consequences and effect of weight loss. J. Intern. Med..

[14] Menendez A., Wanczyk H., Walker J., Zhou B., Santos M., Finck C. (2022). Obesity and adipose tissue dysfunction: from pediatrics to adults. Genes (Basel).

[15] Palacios-Marin I., Serra D., Jiménez-Chillarón J.C., Herrero L., Todorčević M. (2023). Childhood obesity: implications on adipose tissue dynamics and metabolic health. Obes. Rev..

[16] Heier C., Klishch S., Stilbytska O., Semaniuk U., Lushchak O. (2021). The Drosophila model to interrogate triacylglycerol biology. Biochim. Biophys. Acta Mol. Cell Biol. Lipids.

[17] Lehmann M. (2018). Endocrine and physiological regulation of neutral fat storage in drosophila. Mol. Cell Endocrinol..

[18] Gáliková M., Klepsatel P. (2023). Endocrine control of glycogen and triacylglycerol breakdown in the fly model. Semin. Cell Dev. Biol..

[19] Musselman L.P., Kühnlein R.P. (2018). Drosophila as a model to study obesity and metabolic disease. J. Exp. Biol..

[20] Carvalho M., Sampaio J.L., Palm W., Brankatschk M., Eaton S., Shevchenko A. (2012). Effects of diet and development on the Drosophila lipidome. Mol. Syst. Biol..

[21] Church R.B., Robertson F.W. (1966). A biochemical study of the growth of *Drosophila melanogaster*. J. Exp. Zool..

[22] Rodenfels J., Lavrynenko O., Ayciriex S., Sampaio J.L., Carvalho M., Shevchenko A. (2014). Production of systemically circulating hedgehog by the intestine couples nutrition to growth and development. Genes Dev..

[23] Bi J., Xiang Y., Chen H., Liu Z., Grönke S., Kühnlein R.P. (2012). Opposite and redundant roles of the two Drosophila perilipins in lipid mobilization. J. Cell Sci..

[24] Aguila J.R., Suszko J., Gibbs A.G., Hoshizaki D.K. (2007). The role of larval fat cells in adult Drosophila melanogaster. J. Exp. Biol..

[25] Hofbauer H.F., Heier C., Sen Saji A.K., Kühnlein R.P. (2021). Lipidome remodeling in aging normal and genetically obese Drosophila males. Insect Biochem. Mol. Biol..

[26] Musselman L.P., Fink J.L., Ramachandran P.V., Patterson B.W., Okunade A.L., Maier E. (2013). Role of fat body lipogenesis in protection against the effects of caloric overload in drosophila. J. Biol. Chem..

[27] Garrido D., Rubin T., Poidevin M., Maroni B., Le Rouzic A., Parvy J.P. (2015). Fatty acid synthase cooperates with glyoxalase 1 to protect against sugar toxicity. PLoS Genet..

[28] Li S., Yu X., Feng Q. (2019). Fat body biology in the last decade. Annu. Rev. Entomol..

[29] Skowronek P., Wójcik Ł., Strachecka A. (2021). Fat body-multifunctional insect tissue. Insects.

[30] Texada M.J., Koyama T., Rewitz K. (2020). Regulation of body size and growth control. Genetics.

[31] Danielsen E.T., Moeller M.E., Rewitz K.F. (2013). Nutrient signaling and developmental timing of maturation. Curr. Top Dev. Biol..

[32] Tennessen J.M., Thummel C.S. (2011). Coordinating growth and maturation - insights from drosophila. Curr. Biol..

[33] Long W., Wu J., Shen G., Zhang H., Liu H., Xu Y. (2020). Estrogen-related receptor participates in regulating glycolysis and influences embryonic development in silkworm Bombyx mori. Insect Mol. Biol..

[34] Geng D.Q., Wang X.L., Lyu X.Y., Raikhel A.S., Zou Z. (2024). Ecdysone-controlled nuclear receptor ERR regulates metabolic homeostasis in the disease vector mosquito Aedes aegypti. PLoS Genet..

[35] Tennessen J.M., Baker K.D., Lam G., Evans J., Thummel C.S. (2011). The drosophila estrogen-related receptor directs a metabolic switch that supports developmental growth. Cell Metab..

[36] Beebe K., Robins M.M., Hernandez E.J., Lam G., Horner M.A., Thummel C.S. (2020). Drosophila estrogen-related receptor directs a transcriptional switch that supports adult glycolysis and lipogenesis. Genes Dev..

[37] Luo J., Sladek R., Carrier J., Bader J.A., Richard D., Giguère V. (2003). Reduced fat mass in mice lacking orphan nuclear receptor estrogen-related receptor alpha. Mol. Cell Biol..

[38] Carrier J.C., Deblois G., Champigny C., Levy E., Giguère V. (2004). Estrogen-related receptor alpha (ERRalpha) is a transcriptional regulator of apolipoprotein A-IV and controls lipid handling in the intestine. J. Biol. Chem..

[39] Palanker L., Tennessen J.M., Lam G., Thummel C.S. (2009). Drosophila HNF4 regulates lipid mobilization and beta-oxidation. Cell Metab..

[40] Li H., Tennessen J.M. (2017). Methods for studying the metabolic basis of drosophila development. Wiley Interdiscip. Rev. Dev. Biol..

[41] Zirin J., Hu Y., Liu L., Yang-Zhou D., Colbeth R., Yan D. (2020). Large-scale transgenic drosophila resource collections for loss- and gain-of-function studies. Genetics.

[42] Venken K.J., Carlson J.W., Schulze K.L., Pan H., He Y., Spokony R. (2009). Versatile P[acman] BAC libraries for transgenesis studies in Drosophila melanogaster. Nat. Methods.

[43] Palanker L., Necakov A.S., Sampson H.M., Ni R., Hu C., Thummel C.S. (2006). Dynamic regulation of drosophila nuclear receptor activity in vivo. Development.

[44] Öztürk-Çolak A., Marygold S.J., Antonazzo G., Attrill H., Goutte-Gattat D., Jenkins V.K. (2024). FlyBase: updates to the Drosophila genes and genomes database. Genetics.

[45] Grönke S., Mildner A., Fellert S., Tennagels N., Petry S., Müller G. (2005). Brummer lipase is an evolutionary conserved fat storage regulator in drosophila. Cell Metab..

[46] Zinke I., Kirchner C., Chao L.C., Tetzlaff M.T., Pankratz M.J. (1999). Suppression of food intake and growth by amino acids in drosophila: the role of pumpless, a fat body expressed gene with homology to vertebrate glycine cleavage system. Development.

[47] Tennessen J.M., Barry W.E., Cox J., Thummel C.S. (2014). Methods for studying metabolism in drosophila. Methods.

[48] Jensen E.C. (2013). Quantitative analysis of histological staining and fluorescence using ImageJ. Anat. Rec. (Hoboken).

[49] Bradford M.M. (1976). A rapid and sensitive method for the quantitation of microgram quantities of protein utilizing the principle of protein-dye binding. Anal. Biochem..

[50] Matyash V., Liebisch G., Kurzchalia T.V., Shevchenko A., Schwudke D. (2008). Lipid extraction by methyl-tert-butyl ether for high-throughput lipidomics. J. Lipid Res..

[51] Koelmel J.P., Kroeger N.M., Ulmer C.Z., Bowden J.A., Patterson R.E., Cochran J.A. (2017). LipidMatch: an automated workflow for rule-based lipid identification using untargeted high-resolution tandem mass spectrometry data. BMC Bioinformatics.

[52] Pang Z., Lu Y., Zhou G., Hui F., Xu L., Viau C. (2024). MetaboAnalyst 6.0: towards a unified platform for metabolomics data processing, analysis and interpretation. Nucleic Acids Res..

[53] Dainat J. (2024).

[54] Pertea G., Pertea M. (2020). GFF utilities: GffRead and GffCompare. F1000Res.

[55] Patro R., Duggal G., Love M.I., Irizarry R.A., Kingsford C. (2017). Salmon provides fast and bias-aware quantification of transcript expression. Nat. Methods.

[56] Soneson C., Love M.I., Robinson M.D. (2015). Differential analyses for RNA-seq: transcript-level estimates improve gene-level inferences. F1000Res.

[57] Love M.I., Huber W., Anders S. (2014). Moderated estimation of fold change and dispersion for RNA-seq data with DESeq2. Genome Biol..

[58] Zhu A., Ibrahim J.G., Love M.I. (2019). Heavy-tailed prior distributions for sequence count data: removing the noise and preserving large differences. Bioinformatics.

[59] Wilkinson L. (2012). Exact and approximate area-proportional circular Venn and Euler diagrams. IEEE Trans. Vis. Comput. Graph.

[60] Gu Z., Eils R., Schlesner M. (2016). Complex heatmaps reveal patterns and correlations in multidimensional genomic data. Bioinformatics.

[61] Korotkevich G., Sukhov V., Budin N., Shpak B., Artyomov M.N., Sergushichev A. (2021). Fast gene set enrichment analysis. bioRxiv.

[62] Subramanian A., Tamayo P., Mootha V.K., Mukherjee S., Ebert B.L., Gillette M.A. (2005). Gene set enrichment analysis: a knowledge-based approach for interpreting genome-wide expression profiles. Proc. Natl. Acad. Sci. U. S. A..

[63] Dolgalev I. (2022).

[64] Hu Y., Comjean A., Attrill H., Antonazzo G., Thurmond J., Chen W. (2023). PANGEA: a new gene set enrichment tool for drosophila and common research organisms. Nucleic Acids Res..

[65] Consortium T.G.O., Aleksander S.A., Balhoff J., Carbon S., Cherry J.M., Drabkin H.J. (2023). The gene ontology knowledgebase in 2023. Genetics.

[66] Canavoso L.E., Jouni Z.E., Karnas K.J., Pennington J.E., Wells M.A. (2001). Fat metabolism in insects. Annu. Rev. Nutr..

[67] Gilby A.R. (1965). Lipids and their metabolism in insects. Annu. Rev. Entomol..

[68] Barry W.E., Thummel C.S. (2016). The Drosophila HNF4 nuclear receptor promotes glucose-stimulated insulin secretion and mitochondrial function in adults. Elife.

[69] Yi P., Han Z., Li X., Olson E.N. (2006). The mevalonate pathway controls heart formation in drosophila by isoprenylation of Ggamma1. Science.

[70] Deshpande G., Zhou K., Wan J.Y., Friedrich J., Jourjine N., Smith D. (2013). The hedgehog pathway gene shifted functions together with the hmgcr-dependent isoprenoid biosynthetic pathway to orchestrate germ cell migration. PLoS Genet..

[71] Santos A.C., Lehmann R. (2004). Isoprenoids control germ cell migration downstream of HMGCoA reductase. Developmental Cell.

[72] Barton L.J., Sanny J., Packard Dawson E., Nouzova M., Noriega F.G., Stadtfeld M. (2024). Juvenile hormones direct primordial germ cell migration to the embryonic gonad. Curr. Biol..

[73] BELLÉS X., MARTÍN D., PIULACHS M.D. (2005). The mevalonate pathway and the synthesis of juvenile hormone in insects. Annu. Rev. Entomol..

[74] Deshpande G., Schedl P. (2005). HMGCoA reductase potentiates hedgehog signaling in drosophila melanogaster. Developmental Cell.

[75] Deshpande G., Godishala A., Schedl P. (2009). Ggamma1, a downstream target for the hmgcr-isoprenoid biosynthetic pathway, is required for releasing the Hedgehog ligand and directing germ cell migration. PLoS Genet..

[76] Suh J.M., Gao X., Mckay J., Mckay R., Salo Z., Graff J.M. (2006). Hedgehog signaling plays a conserved role in inhibiting fat formation. Cell Metab..

[77] Zhang J., Liu Y., Jiang K., Jia J. (2020). Hedgehog signaling promotes lipolysis in adipose tissue through directly regulating Bmm/ATGL lipase. Dev. Biol..

[78] Grant J., Saldanha J.W., Gould A.P. (2010). A Drosophila model for primary coenzyme Q deficiency and dietary rescue in the developing nervous system. Dis. Model Mech..

[79] Simcox J., Geoghegan G., Maschek J.A., Bensard C.L., Pasquali M., Miao R. (2017). Global analysis of plasma lipids identifies liver-derived acylcarnitines as a fuel source for brown fat thermogenesis. Cell Metab..

[80] Scholtes C., Dufour C.R., Pleynet E., Kamyabiazar S., Hutton P., Baby R. (2024). Identification of a chromatin-bound ERRα interactome network in mouse liver. Mol. Metab..

[81] Vonolfen M.C., Meyer Zu Altenschildesche F.L., Nam H.J., Brodesser S., Gyenis A., Buellesbach J. (2024). Drosophila HNF4 acts in distinct tissues to direct a switch between lipid storage and export in the gut. Cell Rep..

[82] Audet-Walsh É., Giguére V. (2015). The multiple universes of estrogen-related receptor α and γ in metabolic control and related diseases. Acta Pharmacologica Sinica.

[83] Brown E.L., Hazen B.C., Eury E., Wattez J.S., Gantner M.L., Albert V. (2018). Estrogen-related receptors mediate the adaptive response of Brown adipose tissue to adrenergic stimulation. iScience.

[84] Sopariwala D.H., Rios A.S., Pei G., Roy A., Tomaz Da Silva M., Thi Thu Nguyen H. (2023). Innately expressed estrogen-related receptors in the skeletal muscle are indispensable for exercise fitness. FASEB J.

[85] Wang T., Mcdonald C., Petrenko N.B., Leblanc M., Wang T., Giguere V. (2015). Estrogen-related receptor α (ERRα) and ERRγ are essential coordinators of cardiac metabolism and function. Mol. Cell Biol..

[86] Sakamoto T., Matsuura T.R., Wan S., Ryba D.M., Kim J.U., Won K.J. (2020). A critical role for estrogen-related receptor signaling in cardiac maturation. Circ. Res..

