# Supplementary material for: The Drosophila estrogen-related receptor promotes triglyceride storage within the larval fat body
Source: J Lipid Res. 2025 Apr 25;66(6):100815. doi: 10.1016/j.jlr.2025.100815 (PMC12155637; doi:10.1016/j.jlr.2025.100815)
Supplement: Figure S1 [file mmc12.pdf]

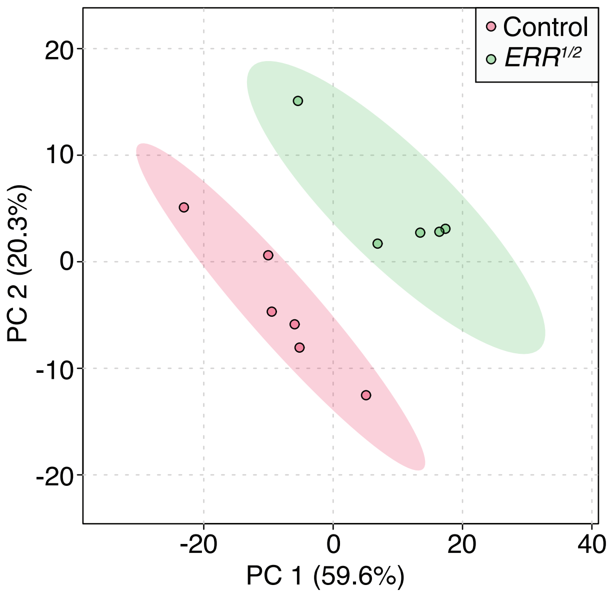

Figure S1. A comparison of the lipidomic data from *ERR$^{1/2}$* mutants and *ERR$^{1/+}$* control samples using principal component (PC) analysis. Targeted metabolomics data from Table S1 was analyzed using principal component analysis. Analysis was conducted using Metaboanalyst 6.0.
