# Supplementary material for: The Drosophila estrogen-related receptor promotes triglyceride storage within the larval fat body
Source: J Lipid Res. 2025 Apr 25;66(6):100815. doi: 10.1016/j.jlr.2025.100815 (PMC12155637; doi:10.1016/j.jlr.2025.100815)
Supplement: Figure S2 [file mmc13.pdf]

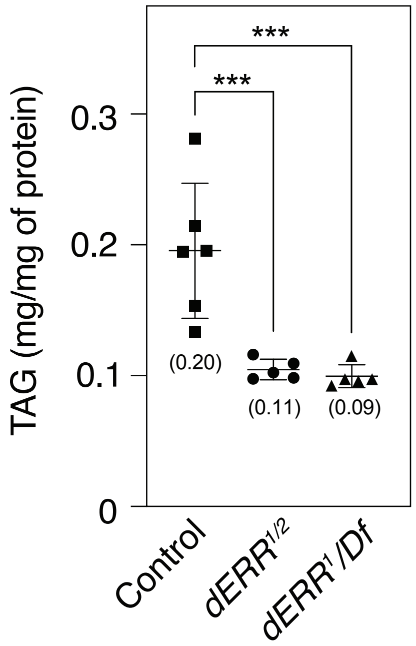

Figure S2. dERR promotes larval TAG accumulation. TAG levels were quantified relative to soluble protein in whole body larval extracts from *dERR*$^{1/+}$ heterozygous controls, *dERR*$^{1/2}$ mutants, and *dERR*$^{1/Df}$ mutants. For *dERR*$^{1/Df}$ mutants the *dERR*^{1} allele was placed in trans to the deficiency *Df(3L)Exel6112*. Data analyzed using an ordinary ANOVA test followed by a Holm-Sidak test for multiple comparisons. ***P<0.001.
