# Supplementary material for: The Drosophila estrogen-related receptor promotes triglyceride storage within the larval fat body
Source: J Lipid Res. 2025 Apr 25;66(6):100815. doi: 10.1016/j.jlr.2025.100815 (PMC12155637; doi:10.1016/j.jlr.2025.100815)
Supplement: Figure S3 [file mmc14.pdf]

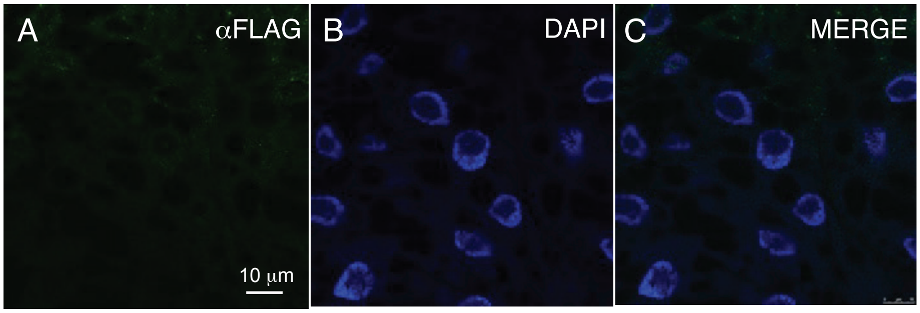

Figure S3. Negative control staining for Figure 2F-H. Fat bodies from *w^{1118}* mid-L2 larvae were stained with αFlag antibody and DAPI. Scale bar in panel (A) applies to (B) and (C).
