# Supplementary material for: The Drosophila estrogen-related receptor promotes triglyceride storage within the larval fat body
Source: J Lipid Res. 2025 Apr 25;66(6):100815. doi: 10.1016/j.jlr.2025.100815 (PMC12155637; doi:10.1016/j.jlr.2025.100815)
Supplement: Figure S4 [file mmc15.pdf]

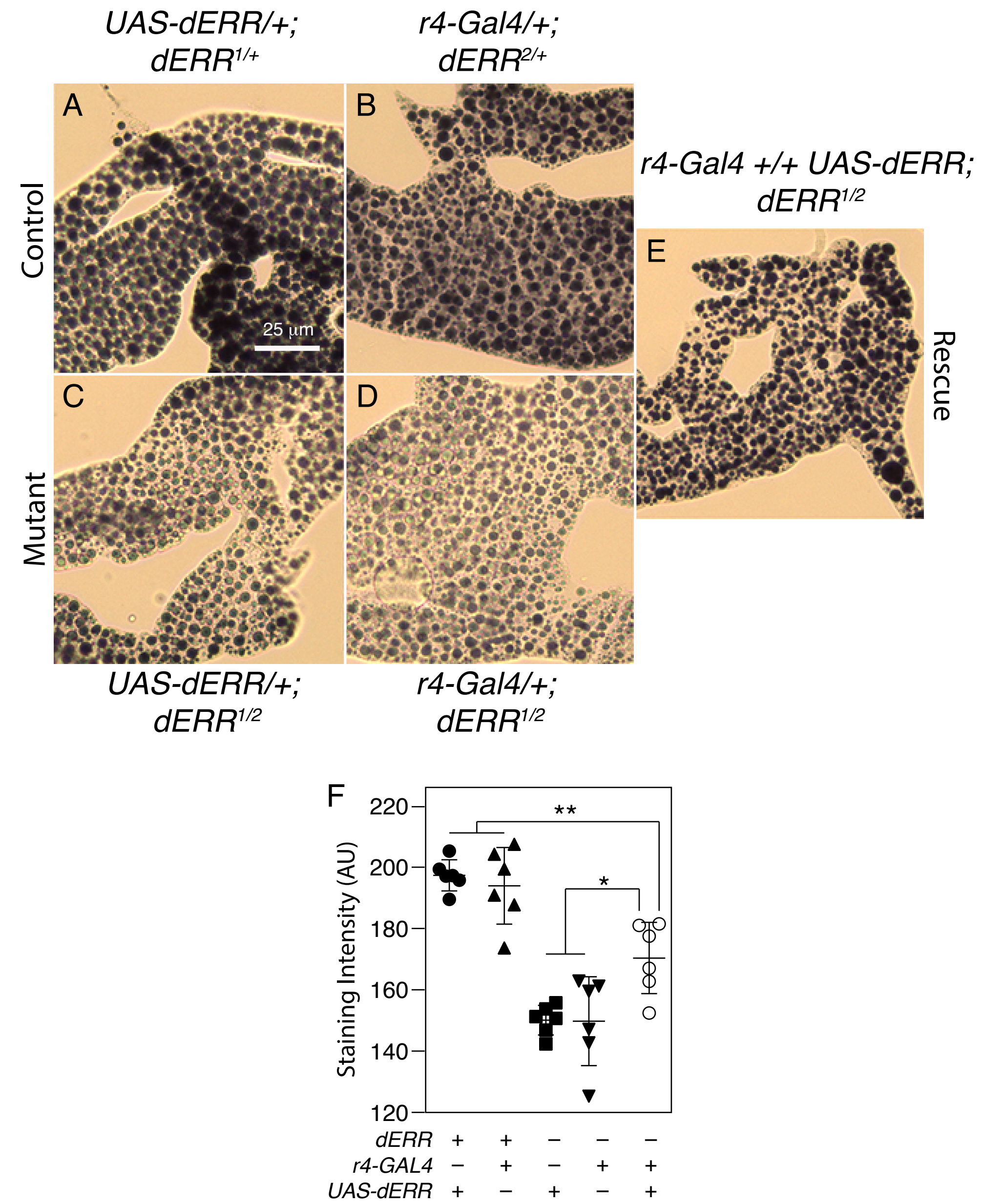

Figure S4. Solvent Black 3 staining of control, *dERR* mutant, and *dERR* rescue larval fat bodies. SB3 staining was used to measure TAG levels in the fat bodies of the heterozygous controls *r4-Gal4*/+; *dERR*$^{2/+}$ and *UAS-ERR*/+; *dERR*$^{1/+}$, the *dERR* mutant controls *r4-Gal4*/+; *dERR*$^{1/2}$ and *UAS-ERR*/+; *dERR*$^{1/2}$, and mutant larvae expressing the rescuing transgene in the fat body (*r4-Gal4* +/+ *UAS-dERR*; *dERR*$^{1/2}$). (A-E) Representative images of SB3 staining for each genotype. (F) Quantification of SB3 staining intensity in L2 fat bodies from heterozygous controls, mutant controls, and rescued larvae (*r4-Gal4* +/+ *UAS-dERR*; *dERR*$^{1/2}$). Scale bar in panel (A) applies to (B-E). Data analyzed using an ordinary ANOVA test followed by a Holm-Sidak test for multiple comparisons. * *P*<0.05. ***P*<0.01.
