# Supplementary material for: The Drosophila estrogen-related receptor promotes triglyceride storage within the larval fat body
Source: J Lipid Res. 2025 Apr 25;66(6):100815. doi: 10.1016/j.jlr.2025.100815 (PMC12155637; doi:10.1016/j.jlr.2025.100815)
Supplement: Figure S5 [file mmc16.pdf]

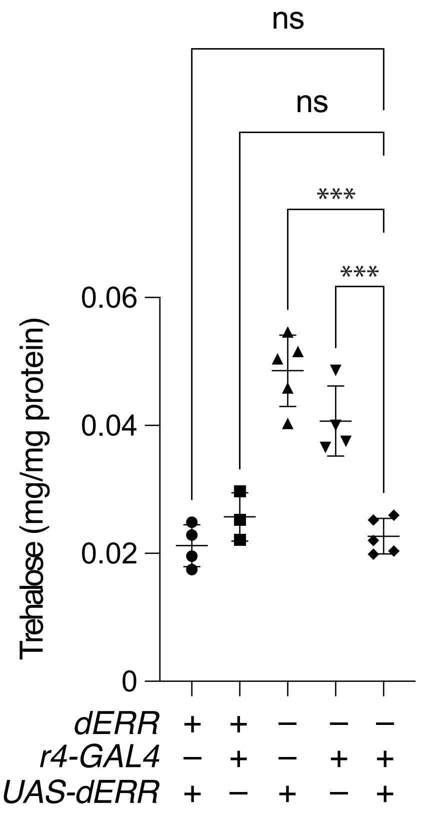

Figure S5. dERR fat body activity regulates systemic trehalose levels. Trehalose levels were quantified relative to soluble protein in whole body extracts from heterozygous controls *r4-Gal4/+*; *dERR*$^{2/+}$ and *UAS-ERR/+*; *dERR*$^{1/+}$, the *dERR* mutant controls *r4-Gal4/+*; *dERR*$^{1/2}$ and *UAS-ERR/+*; *dERR*$^{1/2}$, and mutant larvae expressing the rescuing transgene in the fat body (*r4-Gal4 +/+ UAS-dERR*; *dERR*$^{1/2}$) . Data analyzed using an ordinary ANOVA test followed by a Tukey's multiple comparison test. ***P<0.001.
