# Supplementary material for: The Drosophila estrogen-related receptor promotes triglyceride storage within the larval fat body
Source: J Lipid Res. 2025 Apr 25;66(6):100815. doi: 10.1016/j.jlr.2025.100815 (PMC12155637; doi:10.1016/j.jlr.2025.100815)
Supplement: Figure S6 [file mmc17.pdf]

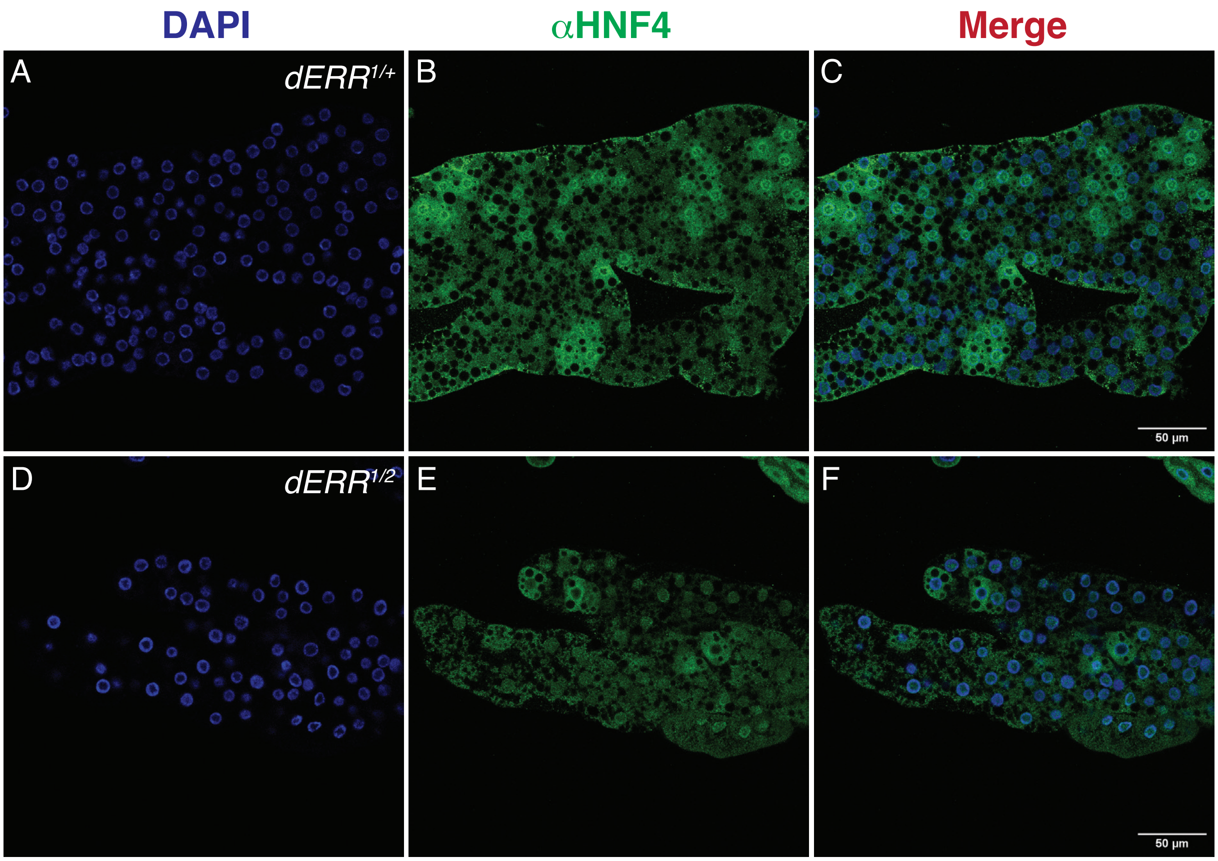


**Figure S6. dHNF4 protein expression in fat bodies from control and dERR mutants.** L2 larval fat bodies were isolated from (A) *dERR*$^{1/+}$ heterozygous controls and (B) *dERR*$^{1/2}$ mutants, fixed, and stained using (A,D) DAPI and (B,E) a previously described dHNF4 antibody (Palanker et al., 2009). The scale bar in panel (C) applies to (A) and (B). The scale bar in panel (F) applies to (D) and (E).
