# Supplementary material for: The Drosophila estrogen-related receptor promotes triglyceride storage within the larval fat body
Source: J Lipid Res. 2025 Apr 25;66(6):100815. doi: 10.1016/j.jlr.2025.100815 (PMC12155637; doi:10.1016/j.jlr.2025.100815)
Supplement: Supplemental Table and Figure Legends [file mmc19.docx]

**SUPPLEMENTAL FIGURE LEGENDS**

**Figure S1. A comparison of the lipidomic data from *ERR^1/2^* mutants and *ERR^1/+^* control samples using principal component (PC) analysis.** Targeted metabolomics data from Table S1 was analyzed using principal component analysis. Analysis was conducted using Metaboanalyst 6.0.

**Figure S2. dERR promotes larval TAG accumulation.** TAG levels were quantified relative to soluble protein in whole body larval extracts from *dERR^1/+^* heterozygous controls, *dERR^1/2^* mutants, and *dERR^1/Df^* mutants. For *dERR^1/Df^* mutants the *dERR^1^* allele was placed in trans to the deficiency *Df(3L)Exel6112*. Data analyzed using an ordinary ANOVA test followed by a Holm-Sidak test for multiple comparisons. ** *P*<0.01. ****P*<0.001.

**Figure S3.** **Negative control staining for Figure 2F-H.** Fat bodies from *w^1118^* mid-L2 larvae were stained with αFlag antibody and DAPI. Scale bar in panel (A) applies to (B) and (C).

**Figure S4. Solvent Black 3 staining of control, *dERR* mutant, and *dERR* rescue larval fat bodies.** SB3 staining was used to measure TAG levels in the fat bodies of the heterozygous controls *r4-Gal4/+*; *dERR^2/+^* and *UAS-ERR/+*; *dERR^1/+^*, the *dERR* mutant controls *r4-Gal4/+*; *dERR^1/2^* and *UAS-ERR/+*; *dERR^1/2^*, and mutant larvae expressing the rescuing transgene in the fat body (*r4-Gal4 +/+ UAS-dERR*; *dERR^1/2^*). (A-E) Representative images of SB3 staining for each genotype. (F) Quantification of SB3 staining intensity in L2 fat bodies from heterozygous controls, mutant controls, and rescued larvae (*r4-Gal4 +/+ UAS-dERR*; *dERR^1/2^*). Scale bar in panel (A) applies to (B-E). Data analyzed using an ordinary ANOVA test followed by a Holm-Sidak test for multiple comparisons. ** *P*<0.01. ****P*<0.001.

**Figure S5. dERR fat body activity regulates systemic trehalose levels.** Trehalose levels were quantified relative to soluble protein in whole body extracts from heterozygous controls *r4-Gal4/+*; *dERR^2/+^* and *UAS-ERR/+*; *dERR^1/+^*, the *dERR* mutant controls *r4-Gal4/+*; *dERR^1/2^* and *UAS-ERR/+*; *dERR^1/2^*, and mutant larvae expressing the rescuing transgene in the fat body (*r4-Gal4 +/+ UAS-dERR*; *dERR^1/2^*) . Data analyzed using an ordinary ANOVA test followed by a Tukey’s multiple comparison test. ****P*<0.001.

**Figure S6. dHNF4 protein expression in fat bodies from control and dERR mutants.** L2 larval fat bodies were isolated from (A) *dERR^1/+^* heterozygous controls and (B) *dERR^1/2^* mutants, fixed, and stained using (A,D) DAPI and (B,E) a previously described dHNF4 antibody (Palanker et al., 2009). The scale bar in panel (C) applies to (A) and (B). The scale bar in panel (F) applies to (D) and (E).

**Figure S7. R scripts used to conduct the RNA-seq processing and RNA-seq analysis.**

**Supplemental Tables**

**Table S1.** Lipidomic analysis of *dERR^1/2^* mutants compared with *dERR^1/+^* heterozygous controls. Samples were collected 60-hours after egg laying (mid L2). Each sample consisted of 20 larvae. Concentrations listed as pmol/mg.

**Table S2.** A list of lipids that exhibit significant changes in *dERR^1/2^* mutants compared with ERR^1/+^ heterozygous controls (absolute FC>2, adjusted *P*<0.1).

**Table S3.** A list of lipids that exhibit significant changes in *dERR^1/2^* mutants compared with *dERR^1/+^* heterozygous controls (absolute FC>1.5, adjusted *P*<0.05).

**Table S4.** RNAseq analysis comparing gene expression in the whole bodies of *dERR^1/2^* mutants with *dERR^1/+^* heterozygous controls. Larvae were collected 60-hours after egg laying (mid L2).

**Table S5.** RNAseq analysis comparing gene expression in fat bodies isolated from *dERR^1/2^* mutants with *dERR^1/+^* heterozygous controls. Larvae were collected 60-hours after egg laying (mid L2).

**Table S6.** Genes that are significantly down-regulated in either Table S4 (*dERR* mutant RNA-seq; whole body) or Table S5 (*dERR* mutant RNA-seq; fat body) are listed according to whether they are down-regulated in only whole animal samples, only fat body samples, or in both sample sets. All genes listed displayed a log2 fold change<-1 and a sval<0.005.

**Table S7.** Genes that are significantly up-regulated in either Table S4 (*dERR* mutant RNA-seq; whole body) or Table S5 (*dERR* mutant RNA-seq; fat body) are listed according to whether they are down-regulated in only whole animal samples, only fat body samples, or in both sample sets. All genes listed displayed a log2 fold change>1 and a sval<0.005.

**Table S8.** PANGEA analysis of significantly down-regulated genes (log2 fold change≤-1 and a sval<0.005) in the fat body of *dERR^1/2^* mutants as compared with *dERR^1/+^* heterozygous controls (see Table S5). PANGEA default settings (FlyBase signaling pathway; SLIM2 GO BP) were used to analyze for enrichment. Only Gene Set IDs with a *P-*value of less than 0.1 are included in the table.

**Table S9.** PANGEA analysis of significantly up-regulated genes (log2 fold change≤-1 and a sval<0.005) in the fat body of *dERR^1/2^* mutants as compared with *dERR^1/+^* heterozygous controls (see Table S5). PANGEA default settings (FlyBase signaling pathway; SLIM2 GO BP) were used to analyze for enrichment. Only Gene Set IDs with a *P-*value of less than 0.1 are included in the table.

**Table S10.** Expression of genes encoding beta-oxidation enzymes in *dERR* mutants.

**Table S11.** A comparison of genes located within 1 kb of dERR and 10 kb of dHNF4 binding sites. The dERR binding sites were identified in Beebe et al 2020. The dHNF4 binding sites were identified in Barry and Thummel 2016. Four genes were found in common between the two studies.
